# Identification of small non-coding RNAs as sperm quality biomarkers for in vitro fertilization

**DOI:** 10.1038/s41421-019-0087-9

**Published:** 2019-04-09

**Authors:** Minmin Hua, Wei Liu, Ying Chen, Fengjuan Zhang, Beiying Xu, Suying Liu, Guowu Chen, Huijuan Shi, Ligang Wu

**Affiliations:** 1 NHC Key Lab of Reproduction Regulation (Shanghai Institute of Planned Parenthood Research), Pharmacy School, Fudan University, Shanghai, 200032 China; 2 State Key Laboratory of Molecular Biology, Shanghai Key Laboratory of Molecular Andrology, CAS Center for Excellence in Molecular Cell Science, Institute of Biochemistry and Cell Biology, Chinese Academy of Sciences, University of Chinese Academy of Sciences, Shanghai, 200031 China; 3 Shanghai Key Laboratory of Female Reproductive Endocrine-Related Diseases, Shanghai Jiai Genetics and IVF Institute, Obstetrics and Gynecology Hospital of Fudan University, Shanghai, 200011 China; 4 Department of In Vitro Fertilization, Shanghai Zhongshan Hospital, Shanghai, 200032 China

**Keywords:** Bioinformatics, Small RNAs

Dear Editor,

In recent decades, assisted reproduction technology (ART) has been widely used to treat human infertility. However, only approximately 30% of in vitro fertilization (IVF) and intracytoplasmic sperm injection (ICSI) cycles result in pregnancy^1^. The classical semen parameters, such as sperm density, morphology, and motility, are not sufficient to effectively assess sperm fertility. Therefore, a method for distinguishing high-quality sperm samples from samples with normal semen parameters is highly desirable. Many types of small non-coding RNAs (sncRNAs), including microRNAs (miRNAs), PIWI-interacting RNAs (piRNAs), tRNA-derived small RNAs (tsRNAs) and rRNA-derived small RNAs (rsRNAs), have been found in mammalian male germ cells and play important regulatory roles in spermatogenesis^2,3^. Identification of the subpopulations of sperm sncRNAs responsible for sperm fertility is of great interest. However, using sncRNAs as prognostic biomarkers for evaluating sperm quality for IVF has not been performed to date. Here, we found that sncRNAs were significantly associated with sperm quality, providing useful biomarkers for improving the success rate of IVF.

In this study, 87 human sperm samples were collected from male partners of couples undergoing IVF treatment (Supplementary Table S1). Microscopic analysis of the isolated sperm by Diff-Quik staining showed that the sperm morphology was normal and the purity was high, which were appropriate for RNA extraction and deep sequencing (Supplementary Fig. S1). The sperm samples were divided into two groups according to the rate of good quality embryos: high rate of good quality embryos (H-GQE, GQE≥75%, *n* = 23) and low rate of good quality embryos (L-GQE, GQE≤25%, *n* = 64)^4^. The maternal influence was excluded as much as possible, and there were no significant differences in age, numbers of oocytes obtained, or MII (metaphase II stage) oocytes among the female partners. The sperm samples were all classified as normal sperm samples by a semen-parameter assessments, including sperm density, morphology, viability and progressive motility^5^, except that the L-GQE group had a slightly higher normal sperm morphology rate (*P* < 0.05) than the H-GQE group.

We extracted total RNAs from 87 human sperm samples and profiled the expression of sncRNAs by deep sequencing. We found that tsRNAs, rsRNAs, miRNAs, and piRNAs were highly abundant in the samples (Fig. 1a; Supplementary Fig. S2a), which is consistent with previous reports in mice^2,3^. On average, ~56% of the sncRNAs annotated to tsRNAs, 18% to rsRNAs, 6% to miRNAs, and 4% to piRNAs (Supplementary Tables S2-S3). The length distribution of these sncRNAs was similar in each sample in both groups. The peak of the tsRNA-, rsRNA-, and miRNA-length ranged from 29 to 34 nt, 17 to 40 nt and 20 to 23 nt, respectively. tsRNAs can be classified into five groups according to the region on tRNAs from which they are derived: 5′-tRNA halves, 3′-tRNA halves, 5′-tRFs (tRNA-derived RNA fragment), i-tRFs (internal tRFs), and 3′-tRFs^6^. Interestingly, among the five types of tsRNAs, the 5′-tRNA halves were the most abundant tsRNAs in human sperm, accounting for more than 75% of all tsRNAs (Supplementary Fig. S2b). rsRNAs can be divided into five types according to the subtypes of rRNA precursors (5S, 5.8S, 18S, 28S, and 45S) from which they are derived^7^. We found that the 28S rRNA precursor-derived rsRNAs (28S rsRNA) were the most abundant rsRNAs, accounting for 60% of all rsRNAs (Supplementary Fig. S2c).

**Fig. 1:**
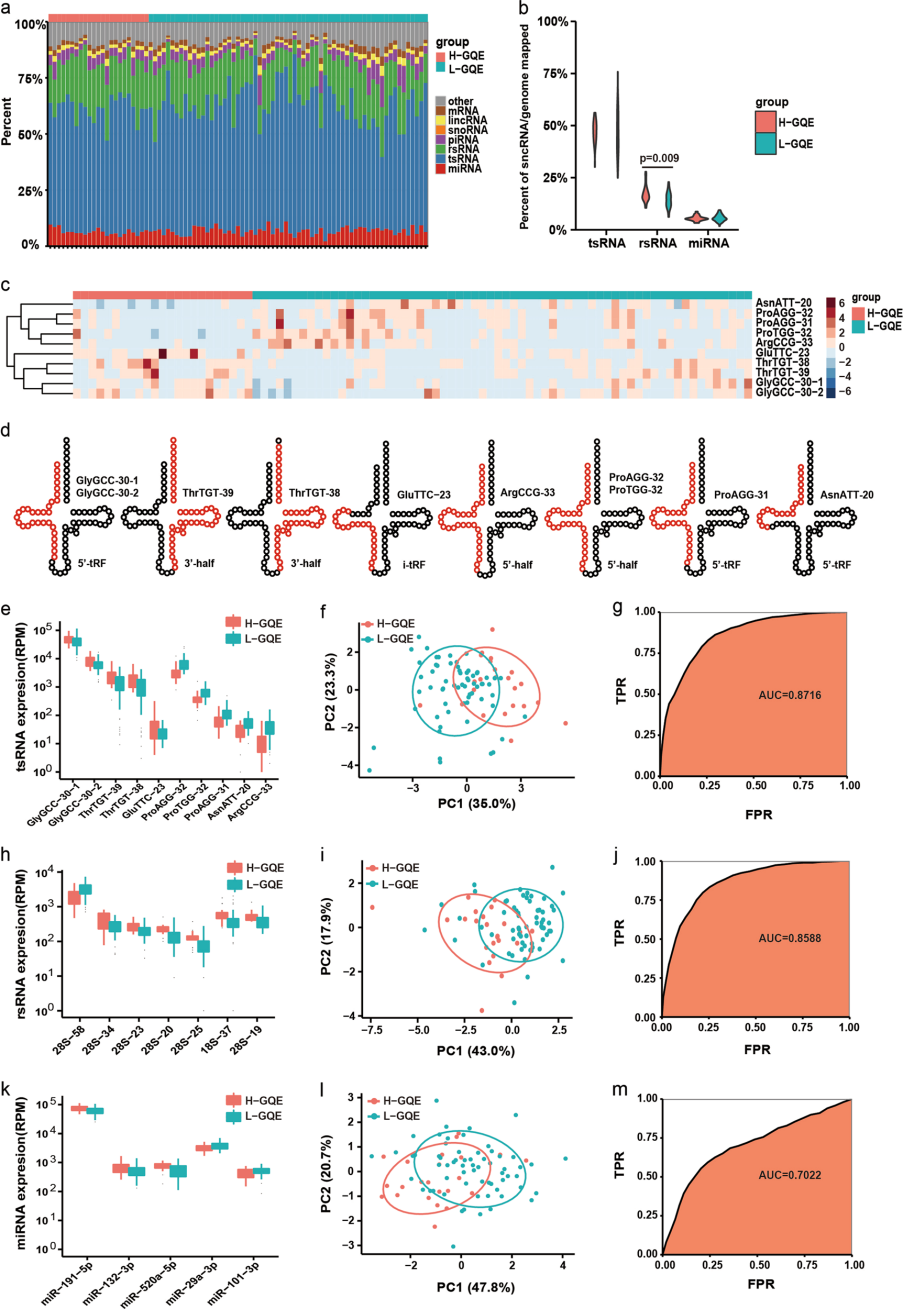
Comparative analysis of sncRNAs in 87 human sperm samples during IVF. **a** Composition of sncRNA categories in 87 human sperm samples. **b** Boxplot of the percentage of tsRNAs, rsRNAs, and miRNAs. The *P*-value of the Wilcoxon signed-rank test is shown in the plot of rsRNA. H-GQE, high rate of good quality embryos; L-GQE, low rate of good quality embryos. **c** Heatmap of ten differentially expressed tsRNAs between the two groups (H-GQE and L-GQE). The branching pattern is illustrated using a dendrogram. **d** A schematic diagram of ten differentially expressed tsRNAs in mature tRNA sequences. tsRNA sequences are highlighted in red. **e** Bar plot of ten differentially expressed tsRNAs between the two groups. **f** Principal component analysis (PCA) of the sperm cells in the two groups based on ten differentially expressed tsRNAs. The variation values of PC1 and PC2 are 35.0% and 23.3%, respectively. Points represent PCA scores of individual samples. Circles represent a general characterization of the PCA space occupied by the ten tsRNAs. Sperm samples from the two groups are shown with different colors. **g** The performance of the support vector machine (SVM) classifier for ten differentially expressed tsRNAs. The area of the receiver operating characteristic curve (AUC) is 0.8716; thus, the classifier is very reliable. TPR: true-positive rate, FPR: false-positive rate. **h** Bar plot of seven differentially expressed rsRNAs between the two groups. **i** PCA of sperm cells in the two groups based on seven differentially expressed rsRNAs. The variation values of PC1 and PC2 are 43.0% and 17.9%, respectively. **j** The performance of the SVM classifier for seven differentially expressed rsRNAs. The AUC is 0.8588. **k** Bar plot of five differentially expressed miRNAs between the two groups. **l** PCA of sperm cells in the two groups based on five differentially expressed miRNAs. The variation values of PC1 and PC2 are 47.8% and 20.7%, respectively. **m** The performance of the SVM classifier for five differentially expressed miRNAs. The AUC is 0.7022

In total, 1899 tsRNAs (average RPM >10) were detected in the sperm samples (Supplementary Table S4). The ratio of total tsRNAs expressed in the L-GQE group was similar to that in the H-GQE group (Fig. 1b). Notably, most of the top 100 tsRNAs were from the 5′-end of the tRNA, including 5′-halves and 5′-tRF (Supplementary Fig. S3a). In contrast, 3′-halves, 3′-tRFs, and i-tRFs were expressed at very low levels. Previous studies showed that 5′-tsRNAs can regulate translation by different mechanisms, such as interfering with translation initiation and RNA modification, or repressing translation by miRNA-like mechanism^8^. Moreover, sperm tsRNAs are able to mediate the transmission of paternal traits to offspring and influence embryonic gene expression in mice^9–11^, indicating that tsRNAs might play important roles in sperm maturation as well as early embryonic development. To further investigate the relationship between tsRNAs and sperm quality, we identified ten differentially expressed tsRNAs between the two groups (Fig. 1c, d; Supplementary Table S5). Among these tsRNAs, five tsRNAs, including GlyGCC-30-1 (the first number indicates the length of the tsRNA, the second number indicates the isoform of tsRNAs with the same length), GlyGCC-30-2, ThrTGT-38, ThrTGT-39, and GluTTC-23, were downregulated in the L-GQE group. Five other tsRNAs, including ProAGG-32, ProTGG-32, ProAGG-31, AsnATT-20, and ArgCCG-33, were upregulated (Fig. 1d, e). These differentially expressed tsRNAs belong to different types of tsRNAs: three were 5′-tRNA halves, two were 3′-tRNA halves, four were 5′-tRFs and one was an i-tRF. Furthermore, we found that a principal component analysis (PCA), which is a powerful tool for exploratory data analysis and generating predictive models, could separate the H-GQE group from the L-GQE group based on these ten tsRNAs (Fig. 1f). The accuracy of the support vector machine (SVM) classifier was evaluated by the area under the ROC curve (AUC), and the results showed the reliability of the classifier for the ten tsRNAs (Fig. 1g, AUC = 0.8716). These results indicated that the ten tsRNAs have an excellent prognostic value and can be potential biomarkers for assessing human sperm quality for IVF. Recently, tRF-Gly-GCC was shown to repress genes associated with the endogenous retroelement MERVL in zygotes and later in development^10^. Intriguingly, we found that two types of 5′-tRF, GlyGCC-30-1, and GlyGCC-30-2, were downregulated in the L-GQE group. Thus, the mis-regulation of these tsRNAs in sperm might contribute to poor sperm quality and abnormal early embryo development, a notion which warrants future investigation.

rsRNAs are a type of rRNA-derived sncRNA abundantly expressed in mature sperm. Previous studies showed that they were sensitive to the environment and were diminished in the sperm of leukocytospermia patients^3,11^. Remarkably, we found that rsRNAs were highly expressed in human sperm (Fig. 1a, b; Supplementary Table S6), especially the rsRNAs derived from the 28S rRNA (Supplementary Fig. S2c, 3b). The ratio of total rsRNAs expressed in the L-GQE group was slightly lower than that in the H-LEQ group (Fig. 1b). To explore the association between rsRNAs and sperm quality, we analyzed the expression of rsRNAs and identified seven differentially expressed rsRNAs: 28S-58, 28S-34, 28S-23, 28S-20, 28S-25, 18S-37, 28S-19 (the number after the dash indicates the length of the rsRNA) (Fig. 1h; Supplementary Table S7). Only 28S-58 was upregulated in the L-GQE group, the remaining six rsRNAs were all downregulated (Fig. 1h). PCA and SVM classifier analyses showed that these seven rsRNAs could also classify the samples into two groups (Fig. 1i, j, AUC = 0.8588), indicating that these rsRNAs have comparable predictive power as tsRNAs and may be another type of useful biomarker for the clinical evaluation of sperm quality.

miRNAs were also expressed in human sperm (Fig. 1a, b), and 467 known miRNAs were detected (average RPM > 10) in 87 samples (Supplementary Table S8). Similar to tsRNAs, there was no significant difference in the ratio of total miRNAs expressed in the two groups (Fig. 1b). Nearly half of the top 100 miRNAs were upregulated in almost half of the L-GQE sperm samples, while another half of the miRNAs were downregulated (Supplementary Fig. S3c), indicating that miRNA expression in these samples is dysregulated during sperm maturation. We identified five miRNAs that were differentially expressed between the two groups; three miRNAs (miR-132-3p, miR-191-3p, and miR-520a-5p) were downregulated, and two miRNAs (miR-101-3p and miR-29a-3p) were upregulated in the L-GQE group (Fig. 1k; Supplementary Table S9). Both PCA and SVM (AUC = 0.7022) analyses showed that the miRNAs can be used for separating the H-GQE and L-GQE groups (Fig. 1l, m). Moreover, an analysis of significant gene ontology (GO)-enriched terms showed that both the downregulated and upregulated target genes were involved in the biological processes of cell development and differentiation (Supplementary Fig. S4a, b), indicating that these miRNA target genes might be important for spermatogenesis and early embryo development.

In conclusion, we investigated differentially expressed sncRNAs in human sperm as candidate markers for evaluating sperm quality during IVF. We demonstrated that differentially expressed tsRNAs, rsRNAs, and miRNAs are linked to sperm quality according to embryo quality, even though these sperm samples were all considered normal by the traditional semen-parameter assessment. Therefore, the sncRNAs, especially tsRNAs and rsRNAs, may be potential clinical biomarkers for the assessment of sperm quality in IVF.

## Data access

All data used to obtain the conclusions in this paper are presented in the paper and Supplementary Information. The deep sequencing data have been deposited in the National Center for Biotechnology Information Expression Omnibus (GEO) (http://www.ncbi.nlm.nih.gov/geo/) under accession number GSE110190.

## Supplementary information

Supplementary Tables, Figures and Methods

Supplementary Table S3-S9

## Supplementary information

**Supplementary Information** accompanies the paper at (10.1038/s41421-019-0087-9).
