# Supplementary material for: Identification of small non-coding RNAs as sperm quality biomarkers for in vitro fertilization
Source: Cell Discov. 2019 Apr 9;5:20. doi: 10.1038/s41421-019-0087-9 (PMC6453904; doi:10.1038/s41421-019-0087-9)
Supplement: Supplementary file 1 — Supplementary Tables, Figures and Methods [file 41421_2019_87_MOESM1_ESM.pdf]

Supplementary Tables, Figures and Methods

Including 9 tables, 4 figures, materials and methods

Supplementary Tables

	Characteristics	H-GQE n=23	L-GQE n=64
Males	Age (years)	35.09±5.98	33.80±4.34
	Sperm density (×10^6/mL)	74.00±25.21	68.89±26.74
	Normal sperm morphology (%)*	14.00±3.90	17.04±6.90
	Sperm viability (%)	58.00±11.66	51.78±14.46
	Grade a+b[a] (%)	43.37±11.78	40.93±12.22
Females	Age (years)	32.57±3.49	32.09±4.24
	No. of obtained oocytes	9.22±3.00	10.09±4.14
	No. of MII oocytes[b]	8.43±3.00	8.16±3.80
Zygotes	No. of 2PN*[c]	7.13±2.80	5.16±3.73
	No. of transferable embryos***	8.41±0.28	3.34±1.16
	Pregnancy rates (%)**	86.80±14.75	64.21±31.31
	Effective embryo rates (%)***	91.29±9.20	33.10±28.03
	Good-quality embryo rates (%)***	87.11±10.40	10.64±10.96

Supplementary Table S1 Characteristics of the participants

H-GQE: high rate of good quality embryos; L-GQE: low rate of good quality embryos.

* $P < 0.05$, ** $P < 0.01$, and *** $P < 0.001$.

[a]: a+b, progressive motility; [b]: MII, metaphase II stage oocytes; [c]: 2PN, zygotes developed to the two pronuclei stage.

sncRNA	Min ratio	Max ratio	Mean ratio
miRNA	2.17%	11.04%	6.72%
tsRNA	32.77%	81.78%	56.50%
rsRNA	6.48%	31.74%	18.44%
piRNA	1.27%	13.82%	4.76%
snoRNA	0.03%	0.45%	0.12%
lincRNA	0.45%	4.38%	1.71%
mRNA	0.91%	4.19%	2.28%
other	4.07%	16.08%	9.47%

Supplementary Table S2 Percentages of the different types of sncRNAs expressed in 87 human sperm samples

Min ratio, Max ratio, Mean ratio: the minimum, maximum and average ratio of each type of sncRNA expressed in 87 human sperm samples.

Supplementary Tables S3-S9 can be found in the file 'Supplementary Tables S3-S9.xslx'.

Supplementary Table S3 Sequencing depth and sncRNA counts

Supplementary Table S4 tsRNA expression profile of 87 samples

Supplementary Table S5 List of differentially expressed tsRNAs according to GQE

Supplementary Table S6 rsRNA expression profile of 87 samples

Supplementary Table S7 List of differentially expressed rsRNAs according to GQE

Supplementary Table S8 miRNA expression profile of 87 samples

Supplementary Table S9 List of differentially expressed miRNAs according to GQE

Supplementary Figures

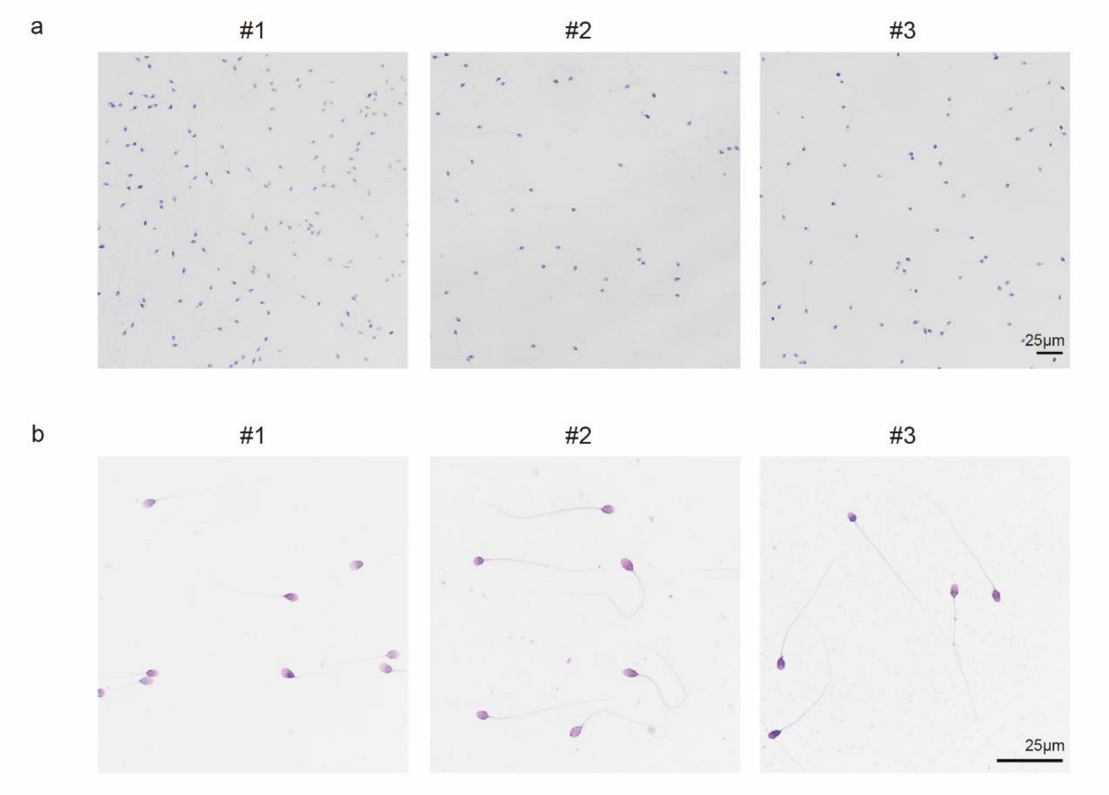

Supplementary Figure S1 Morphology of purified human sperm samples analyzed by Diff-Quik staining. a Purified human sperm under 40× phase contrast microscopy. **b** Purified human sperm under 100× phase contrast microscopy (oil). #1, #2, and #3 correspond to sperm samples from three different patients. Black bar indicates 25 μm.

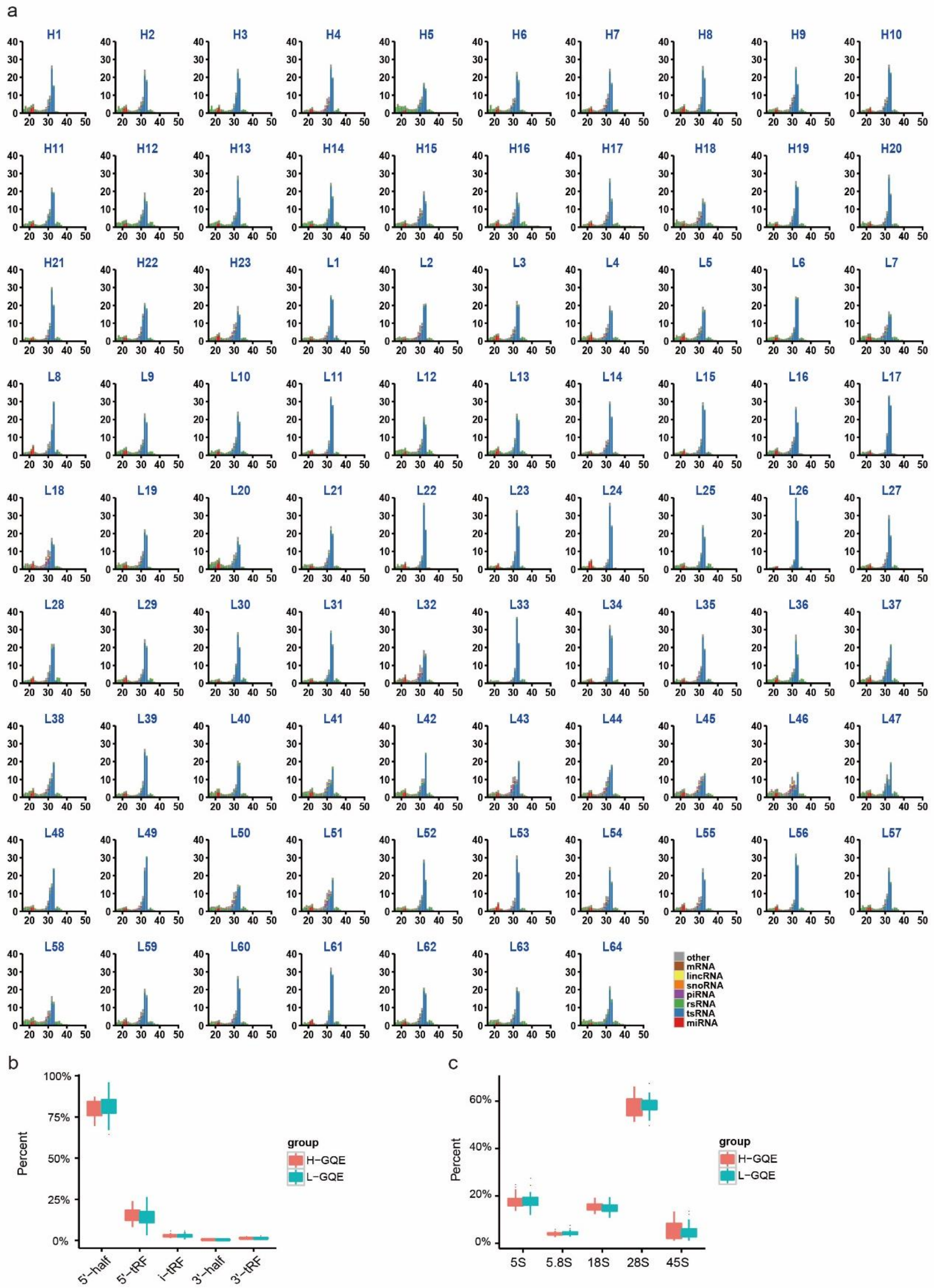

Supplementary Figure S2 Length distribution of sncRNAs and the percentage of different types of sncRNAs. a Length distribution of sncRNAs in 87 human sperm samples. H1-H23: 23 H-GQE samples; L1-L64: 64 L-GQE samples. H-GQE, high rate of good quality embryos; L-GQE, low rate of good quality embryos. **b** The percent of the five types of tsRNAs among all tsRNAs. **c** The percent of the five types of rsRNAs among all rsRNAs. 5S, 5.8S, 18S, 28S, and 45S mean rsRNAs derived from 5S, 5.8S, 18S, 28S, and 45S rRNA, respectively.

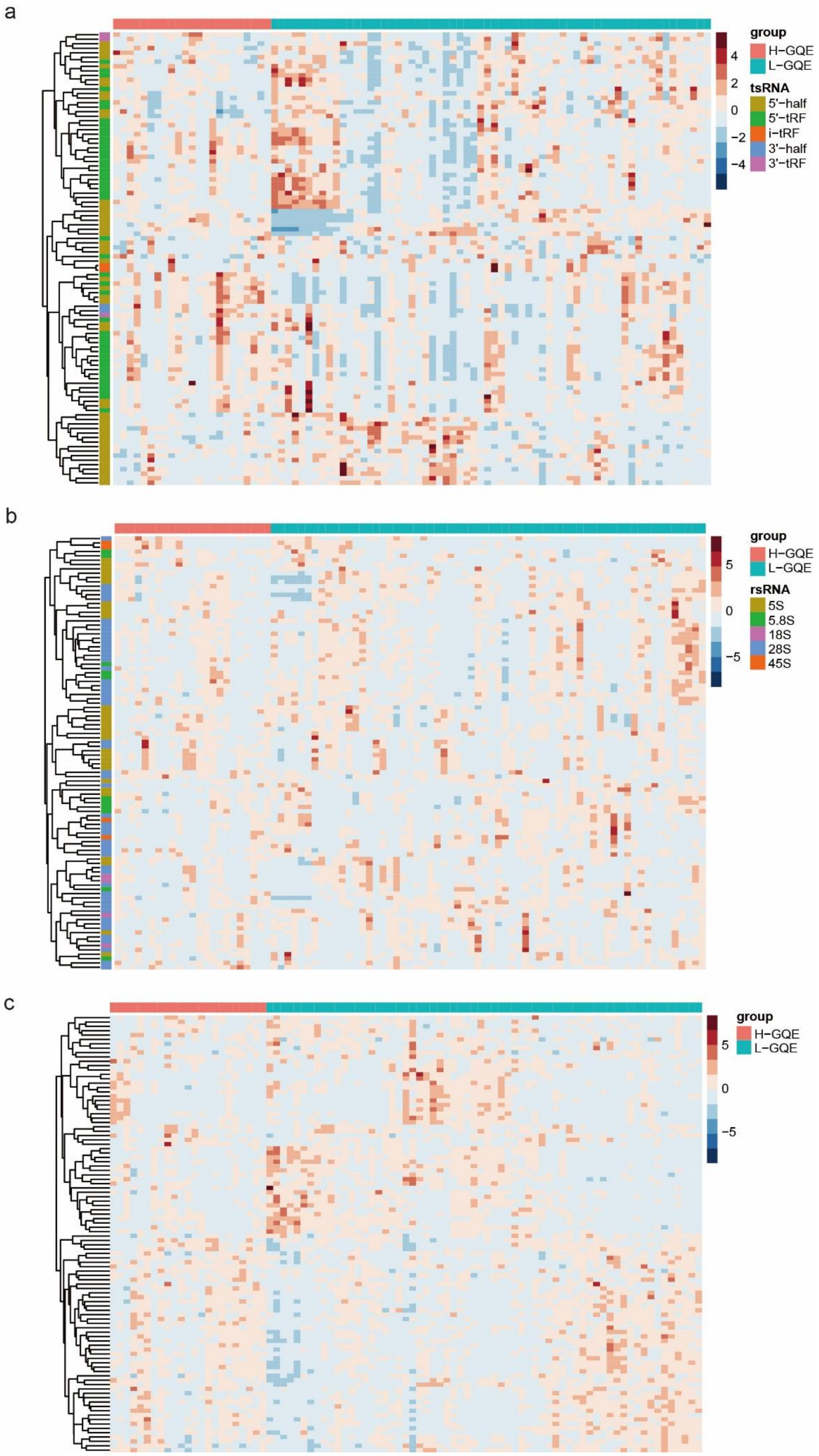
a
group
H−GQE
L−GQE
tsRNA
5'−half
5'−tRF
i−tRF
3'−half
3'−tRF
4
2
0
−2
−4
b
group
H−GQE
L−GQE
rsRNA
5S
5.8S
18S
28S
45S
5
0
−5
c
group
H−GQE
L−GQE
5
0
−5

Supplementary Figure S3 Heatmap of the top 100 sncRNA expression levels. a Heatmap of the top 100 tsRNA expression levels in human sperm samples. Five types of tsRNAs are shown in the heatmap. 5’-half: yellow; 5’-tRF: green; i-tRF: orange; 3’-half: blue; 3’-tRF: pink. **b** Heatmap of the top 100 rsRNA expression levels in human sperm samples. Five types of rsRNAs are shown in the heatmap. 5S: yellow; 5.8S: green; 18S: pink; 28S: blue; 45S: orange. **c** Heatmap of the top 100 miRNA expression levels in human sperm samples. To characterize the changes, the expression of each miRNA between the H-GQE and L-GQE groups was normalized using Z scores. The branching pattern is illustrated with a dendrogram.

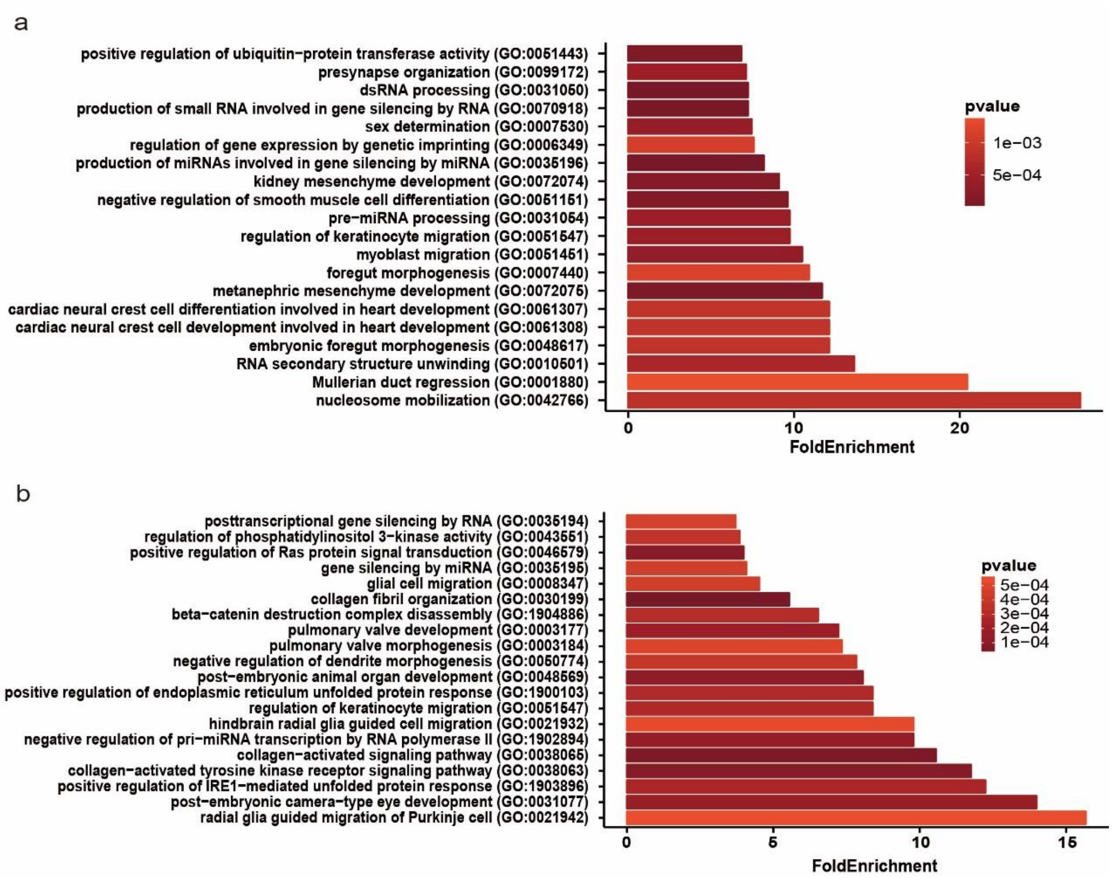

Supplementary Figure S4 GO analysis of the predicted genes targeted by the differentially expressed miRNAs.

a Bar plot showing the Gene Ontology (GO) term categories of the predicted genes targeted by the three downregulated miRNAs (miR-132-3p, miR-191-3p and miR-520a-5p). **b** Bar plot showing the GO term categories of the predicted genes targeted by the two upregulated miRNA (miR-101-3p and miR-29a-3p). The top 20 GO term categories with the highest fold enrichment are shown.

Supplementary materials and methods

Participants and semen samples

In total, 87 couples were recruited for this study between May 2011 and December 2012 at the Shanghai Jiai Genetics and IVF Institute. All couples were of Han nationality and were undergoing their first IVF cycle. The females were aged between 23 and 40 years, and the males were aged between 24 and 50 years. Individuals with any known medical conditions, including diseases not related to fertility, were excluded.

The semen samples were collected by the male participants by masturbation after 3 days of sexual abstinence, allowed to liquefy at 37°C for 30 min, and then processed immediately. Then, the samples were loaded onto 45-90% discontinuous PureSperm gradients (Nidacon International) and centrifuged at 500 g for 20 min at 25°C; next, the top layers were removed. The pellet was washed twice with Ham-F10 medium supplemented with human serum albumin (5 mg/mL) and penicillin G/streptomycin sulfate (0.1 mg/mL; PAN Biotech) and carefully overlaid with 0.75 mL of this supplemented medium. The samples were placed in an incubator at 37°C, and after 45 min, the supernatant was aspirated from the pellet. All semen samples were analyzed for primary semen parameters, including sperm density, motility, viability and morphology, according to the WHO Semen Analysis Manual (5th edition, 2010) [1]. Only normal semen samples were included. We also obtained a phase contrast microscopy image of the purified human sperm using the Diff-Quik staining method[1] to ensure that the purified human sperm had normal morphology with high purity, which is appropriate for RNA extraction and deep sequencing.

Small RNA library preparation and deep sequencing

Total cellular RNA was extracted using TRIzol reagent (Takara). Approximately 200 ng of total cellular RNA was used to construct the small RNA library according to the Illumina protocol. High-throughput RNA sequencing was performed using a Hiseq X Ten (PE150). We used cutadapt to clip adaptor and filter low quality reads [2]. Reads

failing to match the adaptor or reads with lengths shorter than 17 nt were discarded. Redundant sequences were collapsed as useful reads for further analysis [3]. To assess the expression levels of the miRNAs, only reads that exactly matched the 5' start site of the annotated miRNA and the 3' ends with ≤ 2 nt deletions or additional sequences derived from pri-miRNAs were counted as miRNAs. The miRNA count was normalized to the total miRNAs and multiplied by 1,000,000. The expression levels of the tsRNAs and classification were based on MintMAP [4]. The tsRNA count was also normalized to the total tsRNAs and multiplied by 1,000,000. rsRNAs were mapped to rRNA precursors in the order of 5S, 5.8S, 18S, 28S and 45S. The normalized method of rsRNA was same as that of tsRNA and miRNA. The remaining 25-32 nt sequences were used to identify the piRNAs according to a previously described method [5]. sncRNAs with more than one annotation were characterized in the following order: miRNA, tsRNA, rsRNA, snRNA, snoRNA, lncRNA, mRNA and piRNA. Sequences that were not annotated with any of the small RNAs above were classified as other.

Sources of sequences and genome assemblies

The genomic sequences of human (hg38) were downloaded from the University of California Santa Cruz (UCSC) Genome Browser [6] (http://genome.ucsc.edu/). Known RNA sequences were retrieved from the following databases: miRNA, miRbase [7] (version 22.0, http://microrna.sanger.ac.uk/sequences/); tRNAs, Genomic tRNA Database (http://lowelab.ucsc.edu/GtRNAdb/Mmusc); rRNAs, 5.8S, 18S, 28S and 45S from NCBI GenBank (http://www.ncbi.nlm.nih.gov/), 5S from Ensembl (http://www.ensembl.org/index.html); snoRNAs, lncRNAs and mRNAs from Ensembl (http://www.ensembl.org/index.html).

Statistical analysis

We used the R software package for statistical analysis. The differentially expressed tsRNA, rsRNA and miRNA levels were analyzed using DESeq2 [8]. Only sncRNAs with *P*-values less than 0.05 were considered to be differentially expressed sncRNAs

and were used for the subsequent prognostic analysis. Boruta was used for feature selection in the differentially expressed sncRNAs [9]. The Wilcoxon signed-rank test is a statistical significance test that was used to analyze the sncRNA ratio in the two groups (H-GQE and L-GQE); if $P < 0.05$, the association between the tsRNAs/rsRNAs/miRNAs and GQE could be established. Unsupervised hierarchical clustering analysis was conducted using Pheatmap [10].We used the *prcomp* package for the principal component analysis (PCA), which was visualized by ggbiplot [11].

A support vector machine (SVM) classifier was used for this determination to build models based on the 'training' data and searched for similar patterns in the 'testing' data via the SVM function in the e1071 package [12]. We first divided our dataset by random selection into the following two groups: 70% was included in the first group for the SVM classifier training, and the remaining 30% was included in the second group for an independent validation. In addition, we used a receiver operating characteristic (ROC) curve to assess the true positive rate (TPR) of the SVM [12]. The SVM classifiers were trained with the entire first group based on the selected parameters and applied to the second group. The TPR and false positive rate (FPR) of prediction were calculated by setting different thresholds of the SVM score, thus generating a ROC curve that had an area representative of the performance of the SVM classifier. A larger area under the curve (AUC) indicated better performance [13]. To avoid possible bias in the categorization, the assessment described above was repeated 100 times, and the ROC curve was plotted with TPR and FPR averaged from the results of 100 repeated evaluations. The plots were generated using the *ggplot2* package [14].

miRNA functional analysis

The miRNA target genes were predicted by TargetScan (http://www.targetscan.org), which is an online computational algorithm. The predicted target genes of the individual miRNAs were combined as groups of downregulated and upregulated genes. The target gene enrichment test of each miRNA group was annotated based on their biological processes Gene Ontology (GO) terms [15].

References

1 World Health Organization. *WHO laboratory manual for the examination and processing of human semen*. 5th edn, (World Health Organization, 2010).

2 Martin, M. Cutadapt removes adapter sequences from high-throughput sequencing reads. *Embnet Journal* **17** (2011).

3 Gordon, A. & Hannon, G., J. FASTQ/A short-reads preprocessing tools (unpublished) http://hannonlab.cshl.edu/fastx_toolkit. (2010).

4 Loher, P., Telonis, A. G. & Rigoutsos, I. MINTmap: fast and exhaustive profiling of nuclear and mitochondrial tRNA fragments from short RNA-seq data. *Scientific reports* **7**, 41184 (2017).

5 Rosenkranz, D. & Zischler, H. proTRAC--a software for probabilistic piRNA cluster detection, visualization and analysis. *BMC bioinformatics* **13**, 5 (2012).

6 Rosenbloom, K. R. *et al.* The UCSC Genome Browser database: 2015 update. *Nucleic acids research* **43**, D670-681 (2015).

7 Kozomara, A. & Griffiths-Jones, S. miRBase: integrating microRNA annotation and deep-sequencing data. *Nucleic acids research* **39** (2011).

8 Love, M. I., Huber, W. & Anders, S. Moderated estimation of fold change and dispersion for RNA-seq data with DESeq2. *Genome biology* **15** (2014).

9 Kursa, M. B., Jankowski, A. & Rudnicki, W. R. *Boruta - A System for Feature Selection*. (2010).

10 Kolde, R. Pheatmap: pretty heatmaps[J]. *R package version, 61.* (2012).

11 Vu, V. Q. ggbiplot: A ggplot2 based biplot[J]. *R package* (2011).

12 Meyer, D., Dimitriadou, E., Hornik, K., Weingessel, A. & Leisch, F. Misc Functions of the Department of Statistics, ProbabilityTheory Group (Formerly: E1071), TU Wien. (2015).

13 Sing, T., Sander, O., Beerenwinkel, N. & Lengauer, T. ROCR: visualizing classifier performance in R. *Bioinformatics* **21**, 3940-3941 (2005).

14 Wickham., H. & Chang., W. ggplot2: elegant graphics for data analysis. *Springer* (2016).

15 Barrell, D. *et al.* The GOA database in 2009--an integrated Gene Ontology Annotation resource. *Nucleic Acids Res* **37**, D396-403 (2009).
